# Primary extra-nodal non-Hodgkin’s lymphoma affecting mandibular bone: a case report

**DOI:** 10.11604/pamj.2022.41.231.33778

**Published:** 2022-03-22

**Authors:** Julie Gante, Ségolène Georg, Jean-Michel Gabriel Robez, Antoine Dubuc, Frédéric Lauwers

**Affiliations:** 1 Department of Maxillo-Facial Surgery, University Hospital of Toulouse, Paul Sabatier University, Purpan, Toulouse, France,; 2 Dental Surgery, Private Practice, Toulouse, France

**Keywords:** Non-Hodgkin’s lymphoma, mandible, neoplasm, case report

## Abstract

Non-Hodgkin's lymphoma (NHL) is the second most common non-epithelial malignant tumor in the cervicofacial region. Among aggressive NHL, the most common histological type is diffuse large B cell lymphoma (DLBCL). A 44-year-old man presented himself at the oral surgery consultation for the development of isolated dental mobilities associated with mandibular osteolytic lesion. The extraoral examination showed nothing. The neurological examination did not reveal dysesthesia or hypoesthesia. The endo-buccal examination showed an erythematous gum, mobility stage 3 of teeth #42 to 35 with positive pulp sensitivity tests on teeth #34 to 47 and no increased probing depth. The X-rays found homogeneous rounded monogeodic osteolytic lesion extending from teeth #42 to 35 with thinning of the cortical layer. The anatomopathological results of a partial biopsy of the parasymphyseal region found a diffuse large B cell lymphoma of GC phenotype. The patient was referred to the department of oncohematology and treatment was only medical with R-CHOP 21 type immunochemotherapy for 6 cycles. Primary intraosseous localization of non-Hodgkin's lymphoma is rare. The clinical and radiological signs of this malignant tumor pathology are not specific and make its positive diagnosis particularly difficult. In case of uncertainty, an appropriate radiological examination combined with a partial biopsy is essential.

## Introduction

Lymphoid neoplasms include a diverse group of tumors of B-cell, T-cell, and NK-cell origin. They can be classified as Hodgkin's lymphoma (HL) or non-Hodgkin's lymphoma (NHL) [1]. HL is defined by the presence of Reed-Sternberg cells. Non-Hodgkin's lymphoma is more common, with a ratio of 6 to 1, and accounts for 85% of lymphomas. They may affect lymph nodes or other lymphoid organs but they may also have extra-nodal involvement (23 to 30% of NHL) mainly affecting the gastrointestinal tract and the head and neck region [2]. Head and neck lymphomas can develop anywhere and particularly in the Waldeyer's ring [3,4]. Non-Hodgkin's lymphoma in the oral cavity represents 5% of malignant tumors of the head and neck region. In 2008, the World Health Organization classified lymphomas into three groups according to their aggressiveness: indolent, aggressive, or very aggressive. Diffuse large B cell lymphoma (DLBCL) is the most common histological type (60%) [1,5].

## Patient and observation

**Patient information:** a 44-year-old man presented himself at the oral surgery consultation, referred by his dentist for the development of isolated dental mobilities associated with mandibular osteolytic lesion. The significant surgical history shows a sleeve-gastrectomy procedure in 2008 followed by an abdominoplasty in 2016. He has positive history of smoking stopped since 2013, medication and allergic history are insignificant.

**Figure 1:**
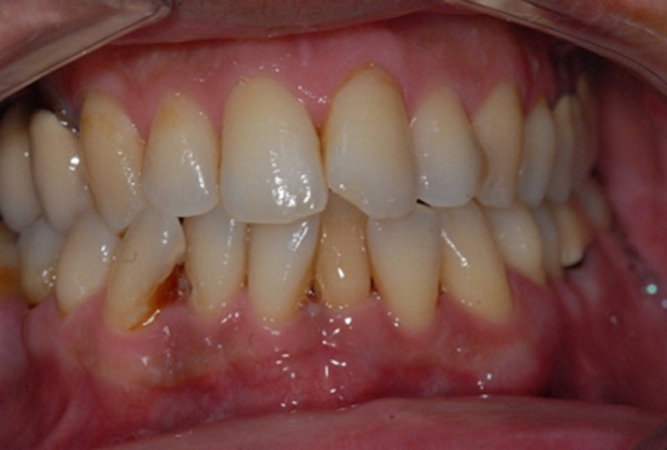
endo buccal examination

**Clinical findings and timeline of current episode:** the history of the disease dates back to 4 months ago when the patient reported an episode of swelling under the left mandible, dysesthesia, as well as a discomfort with chewing leading him to see his dentist. The extraoral examination revealed no swelling, no facial asymmetry or adenopathy. The endo buccal examination (Figure 1) showed a smooth, bright and erythematous gum, especially at the anterior mandibular region with bacterial plaque visible to the naked eye. Pulp sensitivity tests were positive on teeth #34 to 47. The periodontal probing did not find increased probing depth. Teeth #42 to 35 had a mobility of stage 3 on the Mühlemann classification. Palpation of the anterior mandibular vestibule was normal. The neurological examination did not reveal dysesthesia.

**Figure 2:**
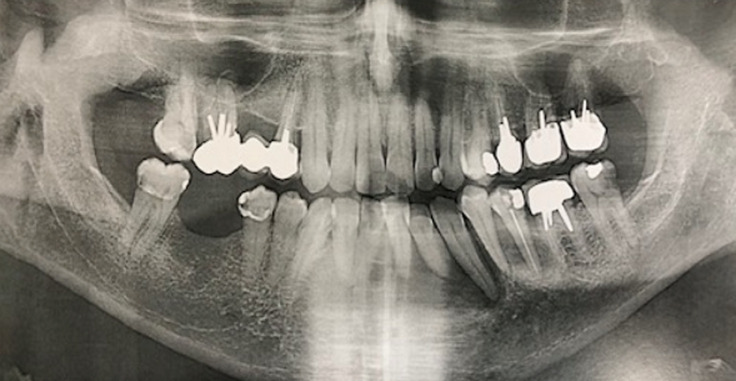
panoramic X-ray: radiolucent mandibular opposite teeth #35 to 42

**Figure 3:**
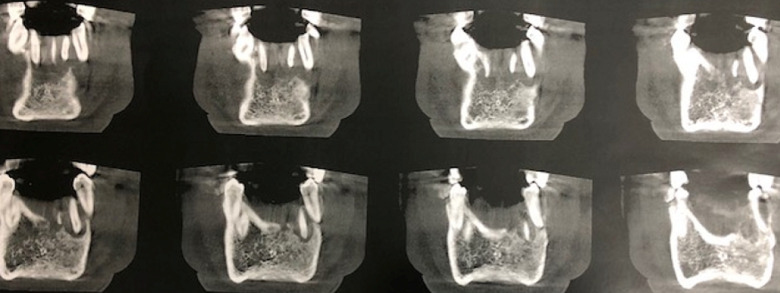
cone beam computed tomography, coronal slices

**Figure 4:**
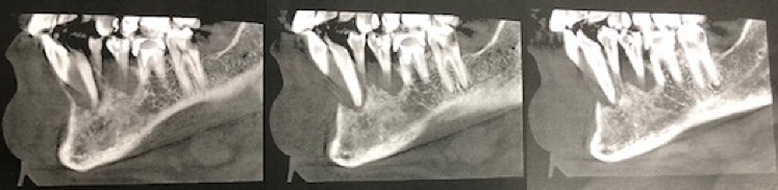
cone beam computed tomography, sagittal slices

**Figure 5:**
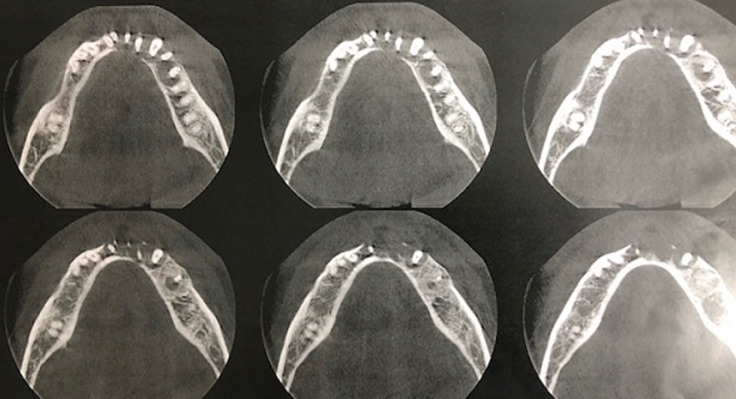
cone beam computed tomography, axial slices: breach of the vestibular cortex and thinning of the lingual cortex

**Diagnostic assessment:** the panoramic X-ray found a well circumscribed radiolucent mandibular image opposite teeth #35 to 42 (Figure 2). There was no peripheral osteocondensation edging. The Cone Beam examination found a homogeneous rounded monogeodic osteolytic lesion extending from teeth #35 to 42 with a breach of the vestibular cortical layer and a thinning of the lingual cortical layer. The left vascular canal was preserved (Figure 3, Figure 4, Figure 5). The clinical picture seemed to suggest a localized periodontal disease. However, the atypical aspect of the radiological image and the mobility of vital teeth without increased probing depth appeared to be compatible with a tumor or cystic lesion. In this context, a partial biopsy of the parasymphyseal region was performed.

**Diagnosis:** the anatomopathological results found a diffuse large B cell lymphoma of GC phenotype (CD 20+, CD 10+, BCL6+, MUM1 negative, KI 67 80%).

**Therapeutic interventions:** the patient was referred to the oncohematology department for further treatment. The biological extension test performed found a normal blood and liver count, protein electrophoresis with no abnormalities, no ionic disorders and negative serology for hepatitis B, hepatitis C and HIV. PET-SCAN mentioned only one bone location (mandibular). The patient presented a diffuse large B cell stage IE lymphoma requiring treatment with R-CHOP 21 type immunochemotherapy (rituximab, cyclophosphamide, vincristine, doxorubicin, methylprednisolone) for 6 cycles.

**Follow up and outcome interventions:** tolerance of the treatment was clinically and biologically optimal from the very first cycles. Six cycles of R-CHOP 21 were carried out in 4 months without any infectious event. The PET-SCAN performed 3 weeks after the last cycle confirmed complete metabolic remission. The patient had regular oncohematological follow-up every 3 months for the first year and then every 6 months. No recurrence has been observed to date. Teeth were treated with local non-surgical periodontal therapy.

**Patient perspective:** “I went to see my dentist for my front teeth that were moving, and I had no idea that I would be diagnosed with oral cancer. I was quickly taken care of and all my chemotherapy cycles went well except for the nausea. Today I continue to have monitoring appointments, but all is well”.

**Informed consent:** an oral informed consent was obtained from the patient.

## Discussion

Diffuse large B cell lymphoma is the most common subtype of NHL, accounting for 30 to 35% of all cases [6]. Oral NHL is rare and usually affects the palate, mandible, oral mucosa, oral floor and gum [7]. The literature suggests preferential localization for the mandibular bone and palate although only 0.6% of NHLs have primary mandibular bone expression [8]. The average age of occurrence is in the 5^th^ decade. Sex ratio is 1.5 men to 1 woman. Some clinical elements may lead to a diagnosis of tumor pathology such as general state alteration, weight loss, night sweats, adenopathies, hepatomegaly, splenomegaly or biological inflammatory syndrome.

Oral NHL can be revealed by pain, swelling, paresthesia or numbness. Others minors clinicals signs such as dental mobility, dental loss or pathological fractures can be observed [4,8,9]. The radiological assessment may reveal an osteolysis around the dental roots or a widening of the chin or mandibular foramen. The symptomatology is aspecific and often leads to diagnostic errors of periapical or periodontal pathologies leading to unjustified therapies (endodontic, tooth extraction, long-term antibiotic therapy). The average delay in diagnosis is about ten weeks [10]. Diagnosis of lymphomas is based on biopsy for which the histopathological examination must be completed by an immunohistochemical examination (labelling with anti-CD20 antibody) [11].

Modified Ann-Arbor classification specifies the body extension at the initial stage [12]. Aggressive NHLs such as DLBCL require the introduction of treatment at any stage at the time of diagnosis. The reference treatment is purely medical and is based on 1^st^ line immunochemotherapy. It combines 6 to 8 cycles of multidrug therapy (CHOP type) with an anti-CD20 monoclonal antibody (rituximab), each cycle being separated by 2 to 4 weeks [5]. This R-CHOP multidrug therapy is the most widely used for stage IE NHL. It shows a significantly increased survival rate of patients with DLBCL compared to CHOP multidrug therapy alone [13].

The prognosis of an LBGDC is assessed according to the International Prognosis Index (IPI) [14]. The etiopathogenesis of LBGDC is not clearly known. Some authors suggest a clonal origin, a chromosomal anomaly, a viral (HIV, EBV) or bacterial (helicobacter pylori) infection, genetic or environmental factors (exposure to toxic substances, radiations) [2,7,11].

## Conclusion

Oral NHL is rare but this presentation illustrates the role of the oral surgeon in early diagnosis of this malignant tumor. Biopsy and rapid referral of the patient to oncohematology department are essential to avoid a delay in management. Management of this challenging disease must be multidisciplinary.
